# Successful olaparib desensitization using a novel one-day protocol

**DOI:** 10.1186/s13223-020-00499-x

**Published:** 2020-11-11

**Authors:** Rongbo Zhu, Stephen Welch, Hannah Roberts

**Affiliations:** 1 Division of Clinical Immunology and Allergy, Department of Medicine, Western University, London, ON Canada; 2 Division of Medical Oncology, Department of Oncology, Western University, London, ON Canada

**Keywords:** Olaparib, Drug desensitization, Drug allergy, Chemotherapy agent

## Abstract

**Background:**

Olaparib is a revolutionary treatment for patients with ovarian and breast cancer. Currently, there is no established 1-day drug desensitization protocol for patients with olaparib type-1 hypersensitivity reactions despite well documented IgE-mediated adverse reactions occurring with olaparib.

**Case presentation:**

We report a 58-year-old female with immediate, reproducible IgE-mediated adverse reactions to olaparib tablets with implementation of a 1-day novel desensitization protocol to olaparib. Following desensitization, the patient was successfully transitioned from olaparib capsules to tablets with no loss of tolerance.

**Conclusions:**

To our knowledge, this is the first reported case of successful olaparib desensitization using a novel 1-day desensitization protocol, and will contribute to drug allergy knowledge, in an area where robust data is lacking. This case demonstrates the important role for drug desensitization in patients with immediate hypersensitivity reactions to chemotherapeutic agents. Furthermore, as olaparib capsules are being phased out in favour of olaparib tablets, we provide a clear case that transitioning from capsule to tablet form did not cause a loss of tolerance.

## Background

Cancer is a leading cause of death in Canada and has a major impact on the health care system [1]. Breast and ovarian cancer are the second and fifth leading cause of cancer related death among Canadian women [1]. Treatments for both cancers generally require aggressive multimodal interventions including surgery, cytotoxic chemotherapy, radiation and biologic therapy [2].

Olaparib is a poly (adenosine diphosphate (ADP)-ribose) polymerase (PARP) inhibitor that is an effective maintenance therapy for patients with platinum sensitive, relapsed ovarian cancer and has provided significant progression-free survival [3]. Following its efficacy in ovarian cancer, it was approved as a novel agent for germline BRCA-mutated, HER2-negative, metastatic breast cancer [4] and very recently, it was approved in North America as a therapy for germline BRCA-mutated metastatic pancreatic cancer [5].

IgE-mediated adverse drug reactions to olaparib are rare, but have been reported [6, 7]. Drug desensitization is a safe and effective way to achieve temporary tolerance in individuals with IgE- mediated reactions to chemotherapy agents to ensure provision of optimal therapy [8, 9]. There is only one report in the literature of successful olaparib desensitization, using a 2-day protocol with olaparib capsules [10]. Maintenance therapy on olaparib capsules (50 mg capsules) results in significant pill burden, as patients typically require 8 capsules twice daily (400 mg twice daily) [7]. In addition, olaparib capsules are being discontinued in some countries, favoring olaparib 150 mg tablets [11].

We describe a patient with significant, reproducible IgE-mediated adverse reactions to olaparib tablets with implementation of a 1-day desensitization protocol to olaparib capsules, with subsequent successful transition from olaparib capsules to tablets.

## Case presentation

A 58-year-old female was referred to the allergy and clinical immunology service from medical oncology for evaluation of olaparib allergy. Her history is significant for recurrent high grade serous ovarian cancer with previous cytoreductive surgery, intraperitoneal chemotherapy and intravenous (IV) chemotherapy (paclitaxel and carboplatin). She was started on 300 mg of olaparib (tablets) and 35 min after her first dose, developed extreme facial flushing and diffuse pruritus. She took diphenhydramine and proceeded with the second dose of olaparib 12 h later. This resulted in immediate lip and facial angioedema, along with pruritus. She administered diphenhydramine and cutaneous symptoms decreased in severity. She continued to take the third dose and within 30 min developed diffuse pruritus, facial urticaria, periorbital and lip angioedema, and nausea. Olaparib was discontinued. There were no symptoms suggestive of a delayed adverse drug reaction. She had no history of chronic spontaneous urticaria and angioedema. She proceeded to take diphenhydramine every 6 h and cutaneous symptoms resolved within 24 h.

Given the patient’s convincing history and reproducible immediate, IgE-mediated symptoms, a Type 1 hypersensitivity reaction to olaparib was diagnosed [8]. As per hospital regulations for the safe handling of cytotoxic medications, epicutaneous testing was not performed at our center. Skin testing was not deemed necessary based on a highly convincing clinical history with objective symptoms witnessed by physicians, non-standardized olaparib skin testing, and the imminent need for olaparib as a first-line therapy.

The patient was admitted as an inpatient and successfully underwent a 9 step, 1-day desensitization protocol we developed (Table 1). As the standard treatment dose of olaparib is 400 mg twice daily, we aimed for a cumulative dose in our desensitization to reflect that. She was pre-medicated with 20 mg of oral cetirizine, 10 mg of montelukast, 50 mg of IV ranitidine, and 10 mg of IV dexamethasone prior to desensitization. During the desensitization, she developed several isolated erythematous macules, but no urticaria, angioedema, or any systemic involvement at a cumulative dose of 450 mg of olaparib capsules. With close monitoring, the desensitization continued to completion with no interruptions and the macules resolved with no intervention required.

**Table 1:** 1-day olaparib desensitization protocol

Steps	Olaparib capsule dose (mg)	Cumulative dose (mg)	Interval since previous dose (minute)	Cumulative duration (Hour:Minute)
1	50	50	–	00:00
2	50	100	30	00:30
3	50	150	30	01:00
4	100	250	60	02:00
5	100	350	60	03:00
6	100	450	90	04:30
7	150	600	90	06:00
8	150	750	120	08:00
9	150	900	120	10:00

The patient successfully continued on oral olaparib capsules with no further adverse reactions. However, given the pill burden of olaparib capsules, she was seen in follow-up as an outpatient 3 weeks later, where she underwent a supervised ingestion to olaparib tablets (Table 2), with no immediate or delayed adverse reaction. The patient has since continued on olaparib tablets without issue and has been monitored in follow-up.

**Table 2:** Supervised ingestion and transition to olaparib tablets

Steps	Olaparib tablet dose (mg)	Cumulative dose (mg)	Interval since previous dose (minute)	Cumulative duration (Hour:Minute)
1	150	150	–	00:00
2	150	300	30	00:30

## Discussion and conclusions

We report the first case of one-day desensitization for an IgE-mediated allergy to olaparib. To our knowledge, there is only one other report of successful olaparib desensitization, using a 2-day protocol with olaparib capsules [5]. One-day drug desensitization protocols are preferred over multiple day protocols as they decrease hospital resources and are more convenient for both the patient and the medical team [9].

There is currently no literature on transitioning from olaparib capsules to tablets after desensitization. Our case is the first to provide evidence that it is safe to transition from olaparib capsules to tablets after desensitization with maintenance of drug tolerance. This becomes increasingly important as olaparib capsules continue to be phased out in favour of olaparib tablets [11].

Our case demonstrates that one-day drug desensitization for olaparib is safe and exemplifies the important role for drug desensitization in patients with immediate hypersensitivity reactions to chemotherapeutic agents. To our knowledge, this is the first reported case of successful olaparib desensitization using a novel 1-day desensitization protocol, and will contribute to drug allergy knowledge, especially chemotherapeutic adverse reactions, in an area where robust data is lacking.

## Data Availability

All data generated or analyzed during this study are included in this published article.

## Abbreviations

ADP: Adenosine diphosphate
BRCA: Breast cancer gene
HER2: Human epidermal growth factor receptor 2
IgE: Immunoglobulin E
IV: Intravenous
PARP: Poly-ADP-ribose polymerase
